# Macrophage Differentiation Selectively Downregulates RPS15A While Preserving RPL13A and RPL17 Expression in THP-1-Derived Macrophages

**DOI:** 10.3390/jcdd13090450

**Published:** 2026-09-09

**Authors:** Joeline Dreik, Nouha Hamoui, Samer Bazzi, Mira Sabat, Zeina Nasr, Jalil Daher

**Affiliations:** 1 Department of Biology, Faculty of Arts and Sciences, University of Balamand, Tripoli 100, Lebanon; joeline.dreik@std.balamand.edu.lb (J.D.); nouha.hamoui@std.balamand.edu.lb (N.H.); 2 Department of Biomedical Sciences, Faculty of Medicine and Medical Sciences, University of Balamand, Tripoli 100, Lebanon; samer.bazzi@balamand.edu.lb; 3 Department of Mathematics, Faculty of Arts and Sciences, University of Balamand, Tripoli 100, Lebanon; mira.sabat@balamand.edu.lb

**Keywords:** atherosclerosis, cardiovascular disease, macrophages, ribosomal proteins, RPS3, RPL13A, RPS15A, RPL17, THP-1

## Abstract

Atherosclerosis is a chronic inflammatory condition that is considered a major contributor to cardiovascular disease (CVD). Atherosclerosis is linked to multiple risk factors, including dyslipidemia, particularly elevated levels of low-density lipoprotein (LDL), which can infiltrate the arterial intima and contribute to disease initiation. Modification of LDL into its oxidized form by myeloperoxidase has been proposed as a physiologically relevant model for LDL oxidation that reflects what happens during the initiation and progression of atherosclerosis. Interestingly, the latter form of oxidized LDL has been shown to induce a myriad of inflammatory reactions within a variety of cells that are involved in atherogenesis. In macrophages (Mφ), accumulation of modified LDL leads to the formation of foam cells which constitute the hallmark of atherosclerosis. Remarkably, Mφs exhibit considerable plasticity and can adopt different phenotypes in response to microenvironmental stimuli. Classically activated M1 macrophages are generally associated with pro-inflammatory responses, whereas alternatively activated M2 macrophages are associated with anti-inflammatory and tissue-repair functions. On the other hand, ribosomal proteins (RPs) have been associated with a range of extra-ribosomal functions that extend beyond their well-recognized role in translation. Notably, multiple RPs, including RPS3, RPL13A, RPS15A and RPL17, have been involved in CVD, playing contrasting roles in the pathogenesis of the disease. In our present study, we investigated, for the first time, the effect of Mφ differentiation, polarization, and Mox-LDL treatment on the expression levels of ribosomal proteins RPS3, RPL13A, RPS15A and RPL17 by using the THP-1 cell model in an effort to reveal potential roles of these ribosomal proteins in Mφ pathobiology. Our study showed that differentiation and polarization significantly downregulate RPS15A expression in THP-1 M0-, M1- and M2-Mφs, whereas differentiation leads to a non-significant trend toward reduced RPS3 expression in M1-Mφs compared with monocytes (*p* = 0.059), while no significant changes in RPL13A and RPL17 expression have been reported under our experimental conditions. These findings indicate that THP-1 monocyte differentiation and macrophage polarization are associated with selective downregulation of RPS15A, whereas the expression of the other investigated ribosomal proteins is largely preserved. Further studies, particularly in primary human macrophages and using functional approaches, are warranted to validate these findings and determine their biological significance and potential relevance to atherosclerosis.

## 1. Introduction

Atherosclerosis, one of the leading causes of global mortality, is a chronic inflammatory disease of the vasculature that severely affects cardiovascular health [1]. It is characterized by the progressive accumulation of lipid-rich plaques containing inflammatory cells, smooth muscle cells, and extracellular matrix within the arterial wall, leading to luminal narrowing and plaque rupture, which can ultimately result in blood flow obstruction and life-threatening complications such as myocardial infarction or stroke [2,3].

Atherosclerosis is driven by a wide range of risk factors that contribute to both disease initiation and progression. These include non-modifiable factors such as age, sex, and genetic background, as well as modifiable factors including dyslipidemia, hypertension, diabetes, obesity, smoking, and alcohol consumption [4,5,6]. A key early event in atherogenesis is the elevation of plasma low-density lipoprotein (LDL), which infiltrates the arterial intima and undergoes oxidative modification to form oxidized LDL (oxLDL) [7]. OxLDL elicits a strong inflammatory response within developing plaques, promoting the recruitment of circulating monocytes into the tunica intima, where they differentiate into Mφs and internalize modified lipoproteins [8]. The excessive accumulation of oxLDL in Mφs leads to the formation of lipid-laden foam cells, a hallmark of atherosclerosis and a major driver of inflammatory signaling during plaque development [9,10,11].

Myeloperoxidase (MPO) has been identified as one of the principal enzymes responsible for LDL oxidation in vivo and is considered a physiologically relevant generator of modified LDL through the production of hypochlorous acid (HOCl) from hydrogen peroxide and chloride ions [11,12]. MPO-oxidized LDL (Mox-LDL) has been implicated in multiple atherogenic processes, including endothelial dysfunction mediated by the upregulation of the LOX-1 scavenger receptor, increased vascular inflammation, and reduced fibrinolytic capacity [13]. Furthermore, Mox-LDL activates monocytes and alters Mφ polarization by increasing tumor necrosis factor-α (TNF-α) secretion and suppressing interleukin-10 (IL-10) expression, without affecting CD209 or CD80 levels, thereby sustaining a pro-inflammatory environment within atherosclerotic lesions [12,14].

Monocytes and Mφs are central components of the innate immune system and play a pivotal role in orchestrating the inflammatory processes underlying atherosclerosis [15]. Their remarkable plasticity allows them to respond to environmental cues and adopt distinct functional phenotypes [16]. Mφs initially differentiate into a non-activated M0 state and subsequently polarize toward a pro-inflammatory M1 phenotype in the presence of lipopolysaccharide (LPS) and interferon-γ (IFN-γ), or toward an anti-inflammatory and tissue-repairing M2 phenotype in response to interleukin-4 (IL-4) and interleukin-13 (IL-13) [17]. Importantly, Mφ polarization varies across different stages of atherosclerosis: M1-Mφs predominate in progressive lesions, where they exacerbate inflammation and destabilize the fibrous cap, whereas M2-Mφs are more abundant in early and stable plaques, where they secrete anti-inflammatory mediators, promote tissue repair, and enhance plaque stability [18].

Ribosomal proteins (RPs) are essential structural and functional components of ribosomes that cooperate with ribosomal RNA to drive mRNA translation into proteins [19]. However, accumulating evidence indicates that RPs possess important extra-ribosomal functions, participating in key cellular processes such as proliferation, differentiation, DNA repair, immune signaling, and apoptosis [20]. Dysregulation of ribosomal proteins has been linked to the development of cancer, metabolic disorders, hematological diseases, and cardiovascular pathologies [21,22]. Several RPs have been implicated in cardiovascular disease progression. For example, RPS3 has been shown to regulate the NLRP3 inflammasome in ischemic stroke, and its silencing promotes a shift in microglial polarization from the pro-inflammatory M1 phenotype to the anti-inflammatory M2 phenotype, suggesting a potential atheroprogressive role for RPS3 [23].

Conversely, RPL13A expressed in Mφs exhibits atheroprotective properties by contributing to the resolution of excessive inflammation [24]. In murine models of atherosclerosis, RPL13A deficiency in Mφs disrupts GAIT-mediated translational silencing, resulting in the upregulation of inflammatory chemokines and chemokine receptors, including CXCL13, CCL22, CCL8, and CCR3, leading to exacerbated inflammation, extensive tissue damage, and reduced survival [25]. In addition, RPS15A has been identified as a key gene associated with venous thromboembolism, a vascular disorder closely linked to cardiovascular disease [26], while RPL17 has been reported to reduce intimal thickening in the carotid arteries of mice, suggesting a protective role in atherosclerosis [27].

Despite the recognized extra-ribosomal functions of RPs and their emerging involvement in inflammatory and cardiovascular processes, little is known about how macrophage differentiation and polarization affect the expression of specific RPs. Accordingly, the present study aimed to investigate whether the expression of RPS3, RPL13A, RPS15A, and RPL17 is altered during THP-1 monocyte differentiation and macrophage polarization and whether Mox-LDL exposure further modulates their expression.

## 2. Material and Methods

### 2.1. Culture of THP-1 Monocytes

The established human monocytic leukemia cell line THP-1 (ATCC TIB-202, Manassas, VA, USA) was kindly provided by Dr. El-Sabban (American University of Beirut) and cultured in complete growth medium consisting of RPMI-1640 medium (#R0883; Sigma-Aldrich, St. Louis, MO, USA), supplemented with 10% heat inactivated FBS (Sigma Aldrich; #F9665), 1% penicillin/streptomycin mixture (#L0022; Biowest, Nuaillé, France), and 1% L-glutamine (#G8540; Sigma-Aldrich, St. Louis, MO, USA). These monocytes were maintained at 37 °C and 5% CO_2_ in a humidified cell culture incubator.

### 2.2. Differentiation of THP-1 Monocytes into M0-Mφs

To prime THP-1 monocytes into M0-Mφs, 6-well culture plates were utilized to seed the cells at a fixed density of 10^6^ cells/well. Cells were then subjected to PMA (phorbol 12-myristate 13-acetate) (#356150050; Thermo Fisher Scientific, Waltham, MA, USA) treatment at a concentration of 100 nM for 48 h. Following this timeframe, PMA-treated cells became adherent. They were washed twice with RPMI-1640 medium (R0883, Sigma-Aldrich, St. Louis, MO, USA) and then maintained for an additional 24 h resting period in a complete growth medium within a humidified incubator. These cells were denoted as M0-Mφs.

### 2.3. Polarization of THP-1 M0-Mφs

M0-Mφs were polarized into M1-Mφs by treatment with recombinant human IFN-γ (Invitrogen by Thermo Fisher Scientific; #RIFNG100) at 20 ng/mL and Ultrapure lipopolysaccharide (LPS) from *Escherichia coli* O111:B4 strain (# tlrl-3pelps; Invitrogen, Thermo Fisher Scientific, Waltham, MA, USA) at 100 ng/mL for 24 h. Alternatively, for M2 polarization, the M0 cells were subjected to treatment with recombinant human IL-13 (#PHC0134; Gibco, Thermo Fisher Scientific, Waltham, MA, USA) at 20 ng/mL and recombinant human IL-4 (#RIL4I; InvivoGen, San Diego, CA, USA) at 20 ng/mL for the same window of time.

#### 2.3.1. Immunophenotyping of THP-1 Monocytes and Mφs

THP-1 monocytes and different Mφ types were collected and incubated at a density of 1 × 10^5^ cells in 100 μL of blocking buffer (DPBS + 2.5% FBS) for 15 min at 4 °C. Cells were then incubated with phycoerythrin (PE)-conjugated mouse anti-human CD11b, allophycocyanin (APC)-conjugated mouse anti-human CD11c, APC-conjugated mouse anti-human CD80, or APC-conjugated mouse anti-human CD209 (BD Biosciences, Franklin Lakes, NJ, USA) for 30 min at 4 °C. Cells were also stained with isotype-matched control antibodies, which included: APC-conjugated IgG1, IgG2a, IgG2b, or PE conjugated mouse IgG1 (BD Biosciences). Following incubation, cells were washed twice with cold DPBS and finally resuspended in 500 µL cold DPBS and run on a flow cytometer within 30 min.

#### 2.3.2. Flow Cytometry Analysis

Surface expression levels of receptors were analyzed on THP-1 monocytes and different Mφ types using a FACSCalibur flow cytometer (BD Biosciences) and CellQuest Pro software 5.1 (BD Biosciences, Franklin Lakes, NJ, USA). Surface receptor expression levels were reported either as percentage of receptor-positive cells or as raw geometric mean fluorescence intensity (MFI) of receptor-positive cells. In addition, forward scatter (FSC) and side scatter (SSC) properties of cells were analyzed. A total of 10,000 single-cell events were measured for each sample.

### 2.4. Mox-LDL Preparation

As previously established and described, Mox-LDL was generated by mixing 1.6 mg of native LDL (#L3486; Invitrogen, Thermo Fisher Scientific, Waltham, MA, USA) (final concentration: 0.8 mg/mL in PBS, pH 7.4), with 45 µL of MPO (#3174-MP; R&D Systems, Minneapolis, MN, USA) (final concentration: 250 nM), 40 µL of hydrogen peroxide (H_2_O_2_) at a concentration of 50 mM (final concentration: 1 mM) and 8 µL of Hydrochloric acid (HCl) at a concentration of 1 M (final concentration: 4 mM). Afterwards, the total final volume was adjusted to 2 mL with DPBS (#D8662; Sigma-Aldrich, St. Louis, MO, USA) (pH 7.4) containing 1 g/L of EDTA [12,28].

### 2.5. Mox-LDL Treatment of Monocytes and M0, M1 and M2-Mφs

Undifferentiated THP-1 monocytes and unpolarized THP-1 M0-Mφs as well as Mφs that were polarized for 24 h into M1 and M2-Mφs were all treated with Mox-LDL at 50 μg/mL for 24 h.

### 2.6. Protein Extraction

To extract proteins, the in-suspension monocytes were centrifuged for 5 min at 1000 rpm at 4 °C. The resulting pellet was resuspended with ice-cold DPBS (Sigma Aldrich; #D8662) for washing and another 5 min of centrifugation was performed at 1200 rpm to pellet the cells. Subsequently, 50 µL of ice-cold lysis buffer (pH 7.5, 25 mM Tris/HCl, 140 mM NaCl, 1% Triton X-100, 1 mM EDTA, 0.5% SDS, 1 mM PMSF, 1 mM Na_3_VO_4_, 1 mM NaF, protease inhibitor cocktail) was added to the pellet and the mixture was incubated on ice for 40 min, while vortexing every 10 min. Finally, centrifugation was performed for 5 min at 13,000 rpm to collect proteins in the supernatant. For adherent Mφs, cells were washed twice with 1 mL of ice-cold DPBS followed by incubation with 100 µL of ice-cold lysis buffer. A scraper was used to detach the adherent cells from the bottom of the wells. Afterward, the plates were gently swirled on ice for 5 min before the lysates were collected and centrifuged at 13,000 rpm for 15 min to isolate the proteins in the supernatant.

### 2.7. Western Blot Analysis

Protein concentration was measured using the Bradford-based protein assay (#500-0006; Bio-Rad Laboratories, Hercules, CA, USA) following the manufacturer’s instructions. For protein separation by SDS-PAGE, gels were prepared as follows: a 4% stacking gel and a 12% resolving gel were formulated. The 4% stacking gel was composed of 30% acrylamide/bis solution (final concentration: 4%), 0.5 M Tris-HCl (pH 6.8) (final concentration: 0.25 M), 10% SDS (final concentration: 0.1%), 60% deionized water, 0.13% TEMED, and 10% APS (final concentration: 0.05%). The 12% resolving gel was prepared with 30% acrylamide/bis solution (final concentration: 12%), 1.5 M Tris-HCl (pH 8.8) (final concentration: 0.375 M), 10% SDS (final concentration: 0.1%), 33% deionized water, 0.1% TEMED, and 10% APS (final concentration: 0.05%). Protein samples were mixed with loading buffer (60% Glycerol, 300 mM Tris pH 6.8, 12 mM EDTA, 12% SDS, 0.05% Bromophenol Blue, distilled water) in a 1:6 ratio and then boiled at 100 °C for 7 min for protein denaturation and separated by electrophoresis at 100 V using 1× Running Buffer (pH 8.3, 0.25 M Tris Base, 1.893 M Glycine, 0.0347 M SDS). Successively, they were transferred onto PVDF blotting membranes (#10600023; Amersham/Cytiva, Marlborough, MA, USA) for 90 min at 100 V and 4 °C, using 1× Transfer Buffer (pH 8.3, 0.25 M Tris Base, 1.92 M Glycine). The membrane was blocked with 15 mL of 5% *w/v* BSA in TBST for 1 h, then incubated overnight, at 4 °C, with the following primary antibodies: anti-RPS3 (#E-AB-60714; Elabscience, Houston, TX, USA) (1:1000), anti-RPL13A (#PA5-17176; Invitrogen, Thermo Fisher Scientific, Waltham, MA, USA) (1:1000), anti-RPS15A (#PA5-87737; Invitrogen, Thermo Fisher Scientific, Waltham, MA, USA) (1:1000), anti-RPL17 (#PA5-29397; Invitrogen, Thermo Fisher Scientific, Waltham, MA, USA) (1:1000) and anti-GAPDH (#MA5-15738; Invitrogen, Thermo Fisher Scientific, Waltham, MA, USA) (1:1000). Subsequently, the membrane was washed in 1× TBST for five separate 10-min washes then incubated with the corresponding secondary antibody for 90 min at room temperature. Ultimately, the membrane was washed again 5 times with 1× TBST for 5 min each and subjected to chemiluminescent detection using Clarity™ western ECL (#170-5061; Bio-Rad Laboratories, Hercules, CA, USA). Chemiluminescent signals were acquired using a ChemiDoc™ MP Imaging System (Bio-Rad Laboratories, Hercules, CA, USA), and densitometric quantification was performed using Image Lab™ Software, version 6.1 (Bio-Rad Laboratories, Hercules, CA, USA). For each sample, the signal intensity of the protein of interest was normalized to the corresponding GAPDH signal. For differentiation and polarization analyses, the normalized values were subsequently expressed relative to the untreated monocyte condition. For densitometric quantification, exposures were selected to ensure that the analyzed signals were unsaturated and within the measurable dynamic range of the imaging system.

### 2.8. Statistical Analysis

Statistical analysis was conducted using GraphPad Prism software (version 10.2.1, San Diego, CA, USA). A two-factor mixed ANOVA was conducted separately for each response variable, with differentiation/polarization and Mox-LDL treatment as factors. The interaction term did not reach statistical significance, and the main effects were therefore examined separately. Marginal ANOVAs were used to assess the effect of differentiation/polarization, while the two-level Mox-LDL treatment factor was assessed using a *t*-test. When the differentiation/polarization effect was significant, Tukey’s post hoc test was performed for pairwise comparisons. Data are presented as the mean ±standard error of the mean (SEM), and a *p*-value < 0.05 was considered statistically significant. “All experiments were performed in at least three independent biological replicates.

## 3. Results

### 3.1. Validation of THP-1 Monocytes Differentiation into M0-Mφs

**Figure 1:**
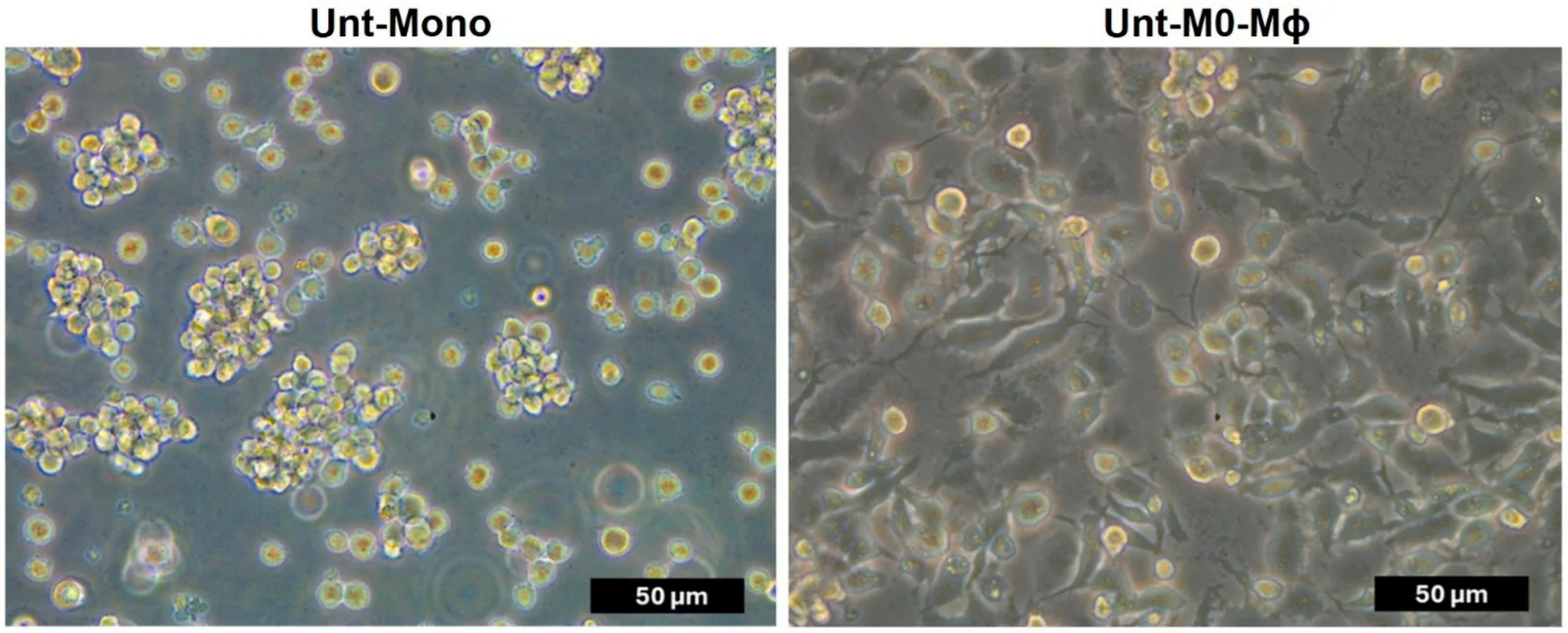
Effect of PMA-Induced Monocyte to Mφ Differentiation on Cell Morphology. Phase-contrast inverted microscopy images of THP-1 monocytes and M0-Mφs at 400× magnification. THP-1 monocytes display a rounded morphology in suspension, while differentiated M0-Mφs exhibit a spindle-like shape post-PMA treatment.

Cellular morphology of THP-1 monocytes was examined using inverted phase contrast microscopy in order to confirm their differentiation into M0-Mφs. Monocytes were in suspension, small in size and grouped together as clusters. Following differentiation, they became adherent, went from round to spindle-like shape, and grew in size, all of which are features of Mφs (Figure 1). This cell line model for differentiation was well-established in multiple previous studies conducted in our lab using the same protocol. For instance, using flow cytometry analysis, our group has demonstrated that treatment of THP-1 monocytes with 100 nM phorbol 12-myristate 13-acetate (PMA) for 48 h followed by consecutive rounds of washing and another period of resting for 24 h leads to their differentiation into M0-Mφs that exhibit increased granularity and upregulated expression of CD11b and CD11c markers on their surface, compared to THP-1 monocytes [14,29] (Supplementary Figures S1 and S2).

### 3.2. Validation of M0 Polarization into M1 and M2-Mφs

**Figure 2:**
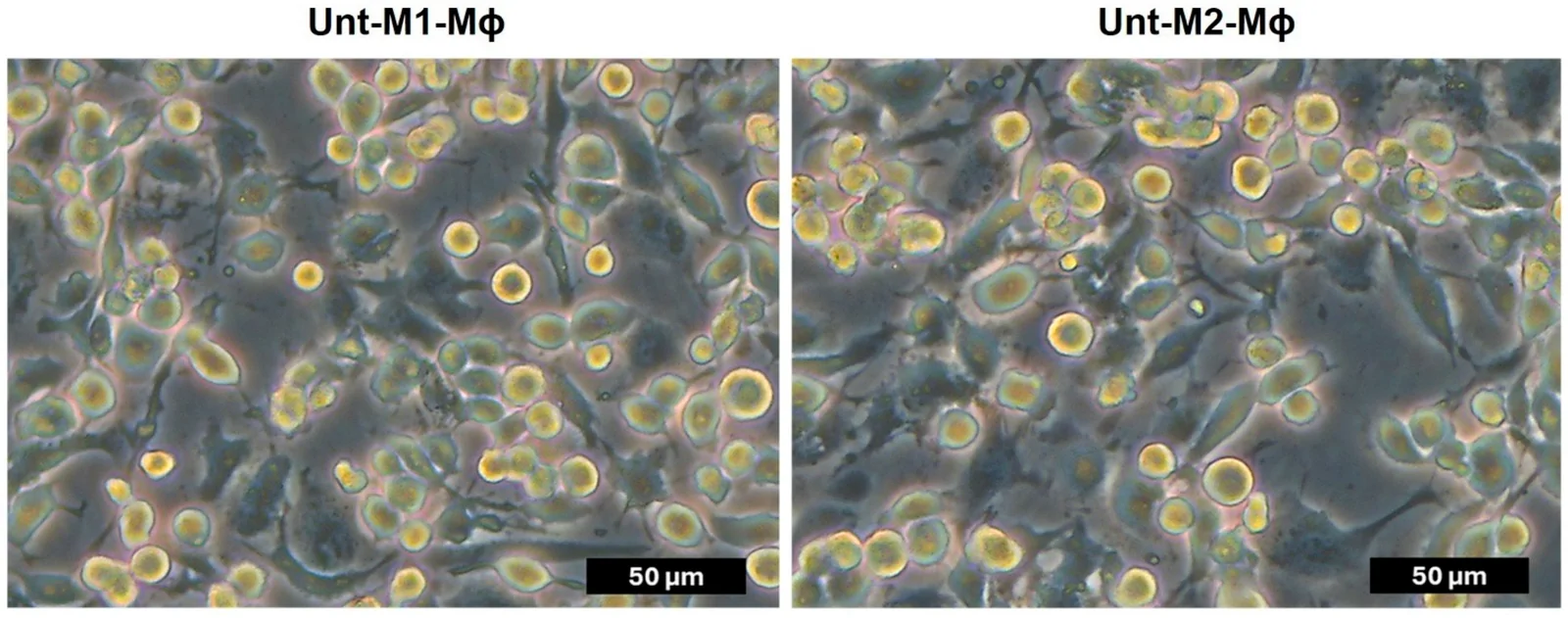
Effect of Polarization on M1 and M2-Mφs Morphology. Phase-contrast inverted microscopy images of THP-1 M1 and M2-Mφs at 400× magnification. M1 and M2-Mφs display a high number of spindle-like shaped cells following polarization.

To confirm M0 polarization into M1 and M2-Mφs, cellular morphology was examined using inverted phase contrast microscopy. The three subtypes of Mφs were adherent. M0-Mφs exhibited a mixture of round and spindle-like shaped cells, with the latter being more dominant. However, M1 and M2-Mφs showed a higher number of spindle-like shaped cells (Figure 2). Again, it is worth mentioning here that, by using flow cytometry analysis and monitoring CD80 (M1 marker) and CD209 (M2 marker) expression, our group has demonstrated that treatment of THP-1 M0-Mφs with IFN-γ (20 ng/mL) and LPS (100 ng/mL) leads to their polarization into M1-Mφs; meanwhile, treating M0-Mφs with IL-13 (20 ng/mL) and IL-4 (20 ng/mL) results in their polarization into M2-Mφs [14,29] (Supplementary Figure S3).

### 3.3. Effect of Differentiation and Polarization on the Expression Level of RPS3

**Figure 3:**
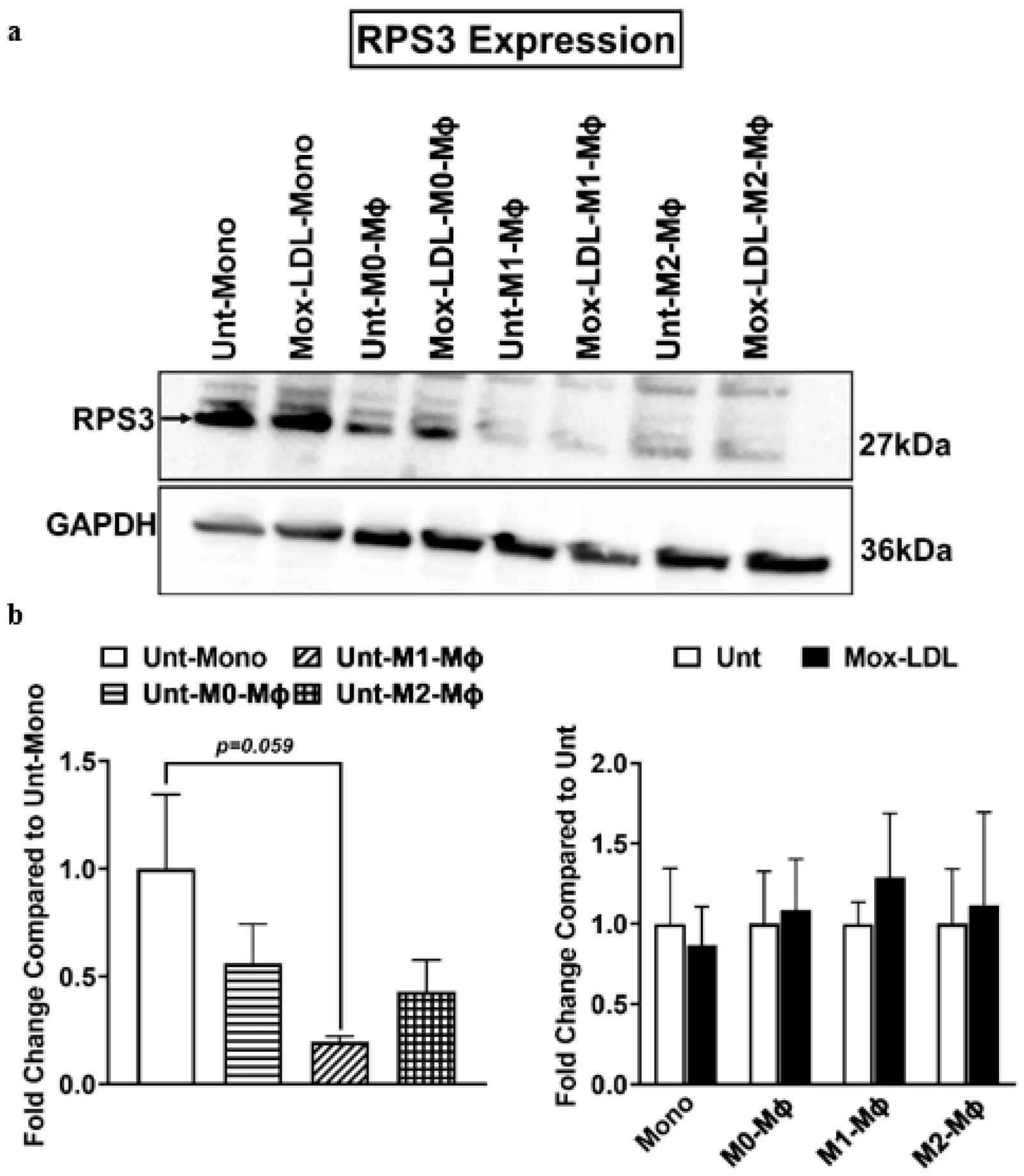
Effect of Differentiation, Polarization and Mox-LDL Treatment on the Expression Level of RPS3. (**a**) Immunoblotting representative results showing the expression level of RPS3 in control untreated and Mox-LDL treated THP-1 monocytes, M0, M1, and M2-Mφs, using GAPDH as a loading control. (**b**) Bar graph (left panel) presenting the relative expression levels of RPS3 in M0, M1, and M2-Mφs compared to monocytes. Data are presented as the mean ± SEM (of 6 independent experiments *n* = 6) fold change in protein expression. Statistical analysis was performed using a two-factor mixed ANOVA. As the interaction between differentiation/polarization and Mox-LDL treatment did not reach statistical significance, main effects were examined separately. The differentiation/polarization effect was assessed using marginal ANOVA followed, when significant, by Tukey’s multiple-comparison post hoc test. The main effect of Mox-LDL treatment was assessed using a *t*-test. Bar graph (right panel) presenting the relative expression levels of RPS3 in Mox-LDL treated THP-1 monocytes, M0, M1, and M2-Mφs compared to their corresponding untreated counterpart subsets. Data are presented as the mean ± SEM (of 6 independent experiments *n* = 6) fold change in protein expression. Unpaired *t*-test was used to calculate statistical significance. Unt-Mono, Untreated monocytes; Unt-M0-Mφs, Untreated M0 macrophage; Unt-M1-Mφs, Untreated M1 macrophage; Unt-M2-Mφs, Untreated M2 macrophage; Mox-LDL-Mono, Mox-LDL-treated monocytes; Mox-LDL-M0-Mφs, Mox-LDL-treated M0 macrophage; Mox-LDL-M1-Mφs, Mox-LDL-treated M1 macrophage; Mox-LDL-M2-Mφs, Mox-LDL-treated M2 macrophage.

Western blotting results showed that RPS3 was expressed in the THP-1 monocytes and the M0, M1, and M2-Mφs (Figure 3). To examine the effect of differentiation and polarization on the expression level of RPS3, Western blot analysis was performed. Although no significant change in RPS3 expression was seen when comparing the M0- and M2-Mφ groups to the monocyte group, there was a non-significant trend toward reduced RPS3 expression in M1-Mφs compared with monocytes (*p* = 0.059) (Figure 3). To examine the effect of Mox-LDL treatment on each cell subset—THP-1 monocytes, M0, M1, and M2-Mφs—we compared the expression levels of RPS3 between 50 μg/mL Mox-LDL treated and untreated (control) groups. No significant differences in protein expression were observed between the treated and control groups in all subsets (Figure 3).

### 3.4. Effect of Differentiation and Polarization on the Expression Level of RPL13A

**Figure 4:**
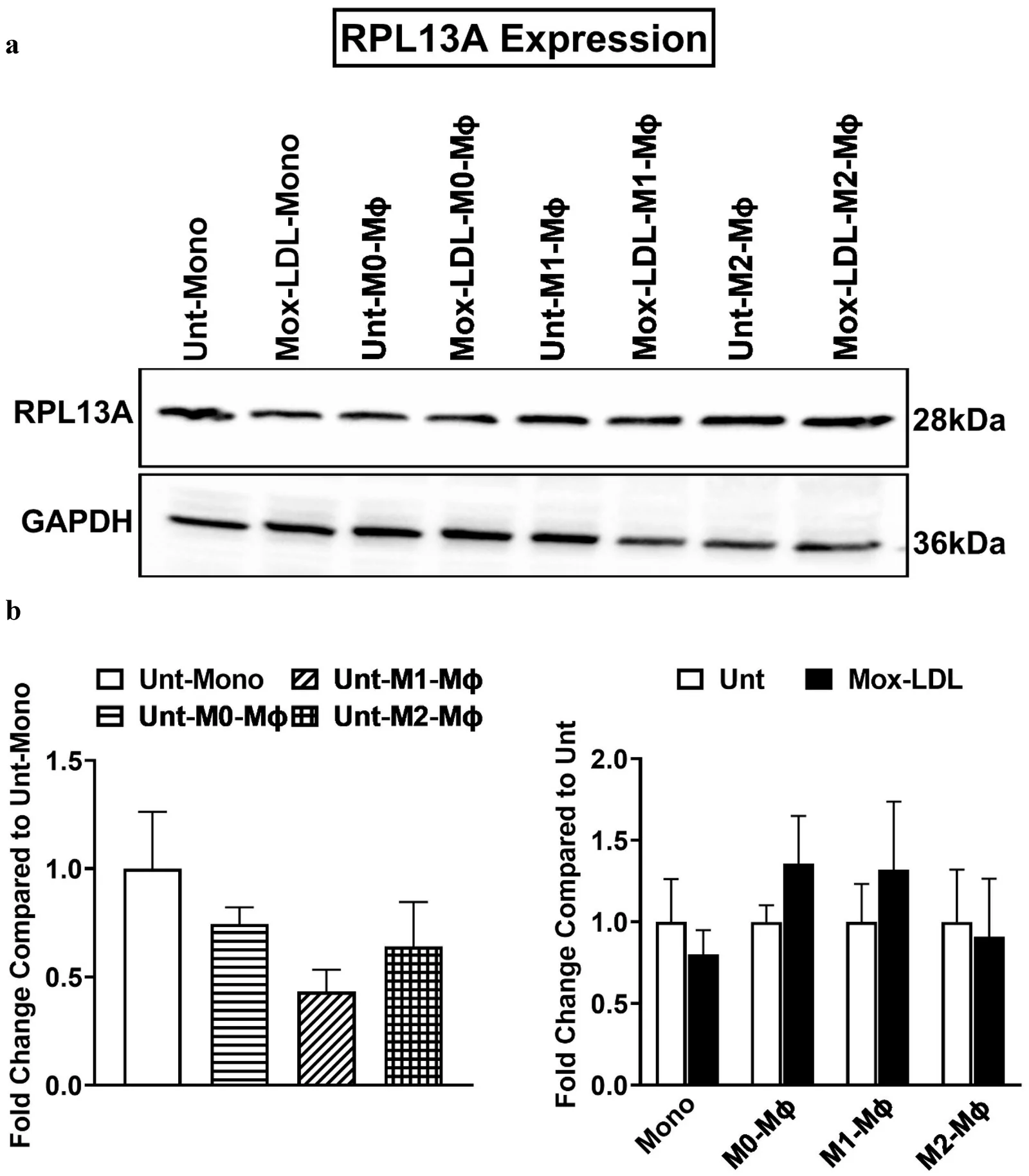
Effect of Differentiation, Polarization and Mox-LDL Treatment on the Expression Level of RPL13A. (**a**) Immunoblotting representative results showing the expression level of RPL13A in control untreated and Mox-LDL treated THP-1 monocytes, M0, M1, and M2-Mφs, using GAPDH as a loading control. (**b**) Bar graph (left panel) presenting the relative expression levels of RPL13A in M0, M1, and M2-Mφs compared to monocytes. Data are presented as the mean ± SEM (of 5 independent experiments *n* = 5) fold change in protein expression. Statistical analysis was performed using a two-factor mixed ANOVA. As the interaction between differentiation/polarization and Mox-LDL treatment did not reach statistical significance, main effects were examined separately. The differentiation/polarization effect was assessed using marginal ANOVA followed, when significant, by Tukey’s multiple-comparison post hoc test. The main effect of Mox-LDL treatment was assessed using a *t*-test. Bar graph (right panel) presenting the relative expression levels of RPL13A in Mox-LDL treated THP-1 monocytes, M0, M1, and M2-Mφs compared to their corresponding untreated counterpart subsets. Data are presented as the mean ± SEM (of 5 independent experiments *n* = 5) fold change in protein expression. Unpaired *t*-test was used to calculate statistical significance. Unt-Mono, Untreated monocytes; Unt-M0-Mφs, Untreated M0 macrophage; Unt-M1-Mφs, Untreated M1 macrophage; Unt-M2-Mφs, Untreated M2 macrophage; Mox-LDL-Mono, Mox-LDL-treated monocytes; Mox-LDL-M0-Mφs, Mox-LDL-treated M0 macrophage; Mox-LDL-M1-Mφs, Mox-LDL-treated M1 macrophage; Mox-LDL-M2-Mφs, Mox-LDL-treated M2 macrophage.

Western blotting results showed that RPL13A was expressed in the THP-1 monocytes and the M0, M1, and M2-Mφs (Figure 4). RPL13A showed no significant difference in its expression level among the four cell subsets (Figure 4). Nonetheless, a downward trend in expression levels was observed among all corresponding groups after differentiation and polarization, but these differences were not statistically significant (Figure 4). To examine the effect of Mox-LDL treatment on each cell subset—THP-1 monocytes, M0, M1, and M2-Mφs—we compared the expression levels of RPL13A between 50 μg/mL Mox-LDL treated and untreated (control) groups. No significant differences in protein expression were observed between the treated and control groups in all subsets (Figure 4).

### 3.5. Effect of Differentiation and Polarization on the Expression Level of RPS15A

**Figure 5:**
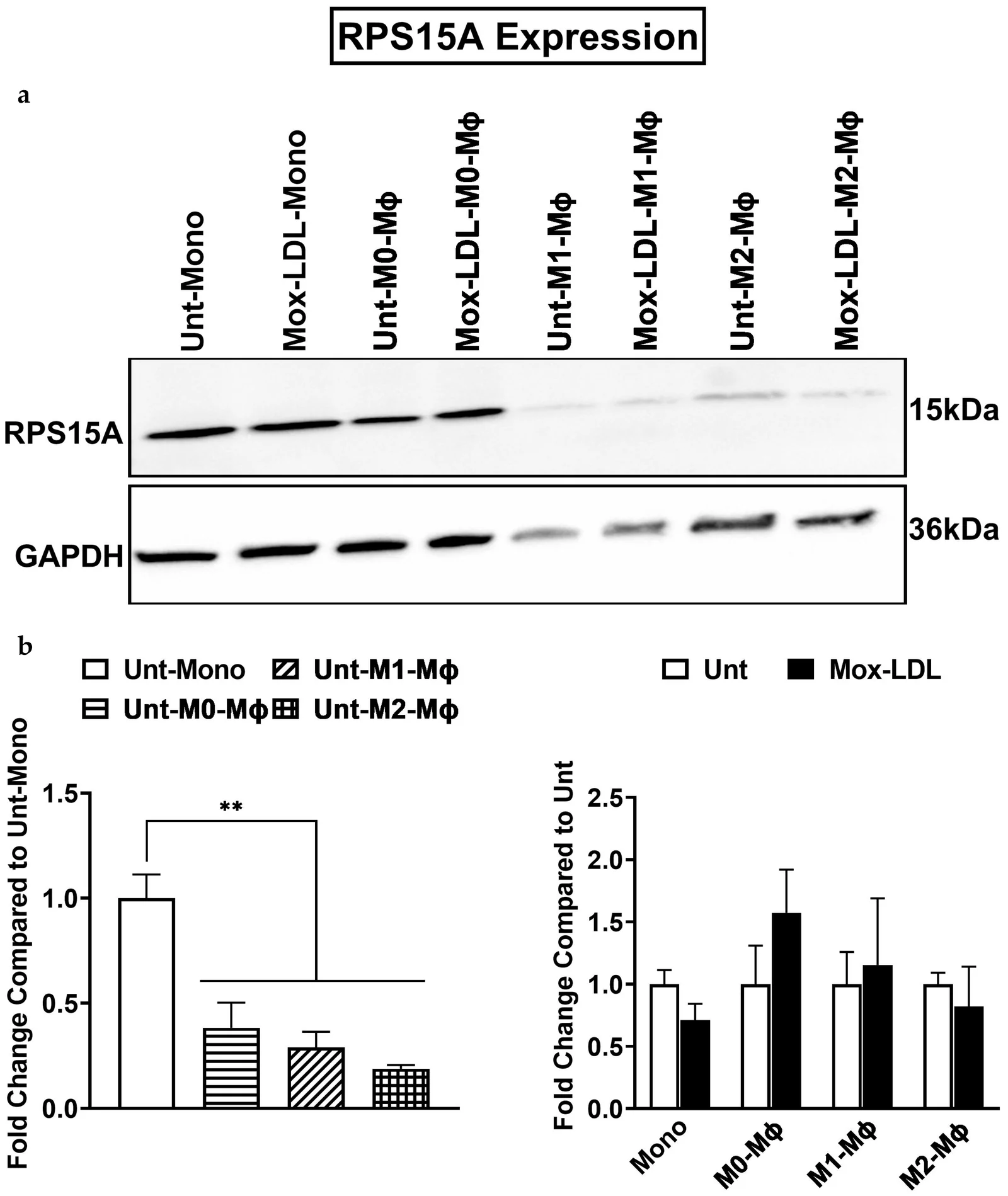
Effect of Differentiation, Polarization and Mox-LDL Treatment on the Expression Level of RPS15A. (**a**) Immunoblotting representative results showing the expression level of RPS15A in control untreated and Mox-LDL treated THP-1 monocytes, M0, M1, and M2-Mφs, using GAPDH as a loading control. (**b**) Bar graph (left panel) presenting the relative expression levels of RPS15A in M0, M1, and M2-Mφs compared to monocytes. Data are presented as the mean ± SEM (of 3 independent experiments *n* = 3) fold change in protein expression. Statistical analysis was performed using a two-factor mixed ANOVA. As the interaction between differentiation/polarization and Mox-LDL treatment did not reach statistical significance, main effects were examined separately. The significant differentiation/polarization effect was further evaluated using Tukey’s multiple-comparison post hoc test. The main effect of Mox-LDL treatment was assessed using a *t*-test. ** *p* < 0.01. Bar graph (right panel) presenting the relative expression levels of RPS15A in Mox-LDL treated THP-1 monocytes, M0, M1, and M2-Mφs compared to their corresponding untreated counterpart subsets. Data are presented as the mean ± SEM (of 3 independent experiments *n* = 3) fold change in protein expression. Unpaired *t*-test was used to calculate statistical significance. Unt-Mono, Untreated monocytes; Unt-M0-Mφs, Untreated M0 macrophage; Unt-M1-Mφs, Untreated M1 macrophage; Unt-M2-Mφs, Untreated M2 macrophage; Mox-LDL-Mono, Mox-LDL-treated monocytes; Mox-LDL-M0-Mφs, Mox-LDL-treated M0 macrophage; Mox-LDL-M1-Mφs, Mox-LDL-treated M1 macrophage; Mox-LDL-M2-Mφs, Mox-LDL-treated M2 macrophage.

Western blotting results showed that RPS15A was expressed in the THP-1 monocytes and the M0, M1, and M2-Mφs (Figure 5). To examine the effect of differentiation and polarizationon the expression level of RPS15A, Western blot analysis was performed. A significant decrease (*p* < 0.01) in RPS15A expression was observed among all M0-, M1- and M2-Mφ groups when compared to the monocyte group (RPS15A expression was 0.384-fold in M0-Mφs, 0.289-fold in M1-Mφs, and 0.189-fold in M2-Mφs relative to monocytes). (Figure 5). These findings indicate that differentiation and polarization are associated with reduced RPS15A expression. To examine the effect of Mox-LDL treatment on each cell subset—THP-1 monocytes, M0, M1, and M2-Mφs—we compared the expression levels of RPS15A between 50 μg/mL Mox-LDL treated and untreated (control) groups. No significant differences in protein expression were observed between the treated and control groups in all subsets (Figure 5).

### 3.6. Effect of Differentiation and Polarization on the Expression Level of RPL17

**Figure 6:**
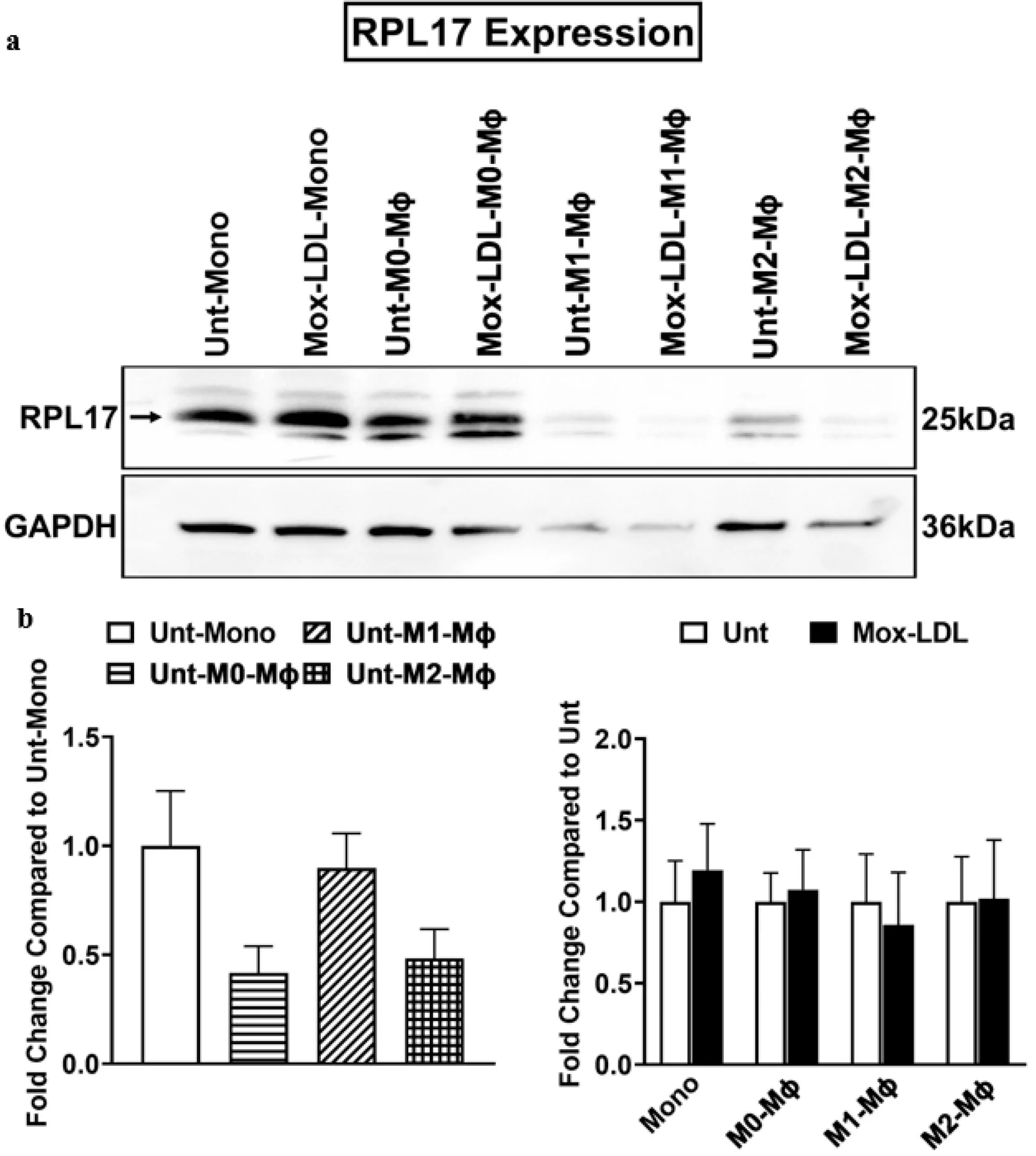
Effect of Differentiation, Polarization and Mox-LDL Treatment on the Expression Level of RPL17. (**a**) Immunoblotting representative results showing the expression level of RPL17 in control untreated and Mox-LDL treated THP-1 monocytes, M0, M1, and M2-Mφs, using GAPDH as a loading control. (**b**) Bar graph (left panel) presenting the relative expression levels of RPL17 in M0, M1, and M2-Mφs compared to monocytes. Data are presented as the mean ± SEM (of 5 independent experiments *n* = 5) fold change in protein expression. Statistical analysis was performed using a two-factor mixed ANOVA. As the interaction between differentiation/polarization and Mox-LDL treatment did not reach statistical significance, main effects were examined separately. The differentiation/polarization effect was assessed using marginal ANOVA followed, when significant, by Tukey’s multiple-comparison post hoc test. The main effect of Mox-LDL treatment was assessed using a *t*-test. Bar graph (right panel) presenting the relative expression levels of RPL17 in Mox-LDL treated THP-1 monocytes, M0, M1, and M2-Mφs compared to their corresponding untreated counterpart subsets. Data are presented as the mean ± SEM (of 5 independent experiments *n* = 5) fold change in protein expression. Unpaired *t*-test was used to calculate statistical significance. Unt-Mono, Untreated monocytes; Unt-M0-Mφs, Untreated M0 macrophage; Unt-M1-Mφs, Untreated M1 macrophage; Unt-M2-Mφs, Untreated M2 macrophage; Mox-LDL-Mono, Mox-LDL-treated monocytes; Mox-LDL-M0-Mφs, Mox-LDL-treated M0 macrophage; Mox-LDL-M1-Mφs, Mox-LDL-treated M1 macrophage; Mox-LDL-M2-Mφs, Mox-LDL-treated M2 macrophage.

Finally, Western blotting experiments were conducted to confirm that RPL17 was expressed in the THP-1 monocytes and the M0, M1, and M2-Mφs (Figure 6). Our analysis showed that the expression pattern of RPL17 was stable in all groups and that it was unaffected by the differentiation and polarization processes (Figure 6). To examine the effect of Mox-LDL treatment on each cell subset—THP-1 monocytes, M0, M1, and M2-Mφs—we compared the expression levels of RPL17 between 50 μg/mL Mox-LDL treated and untreated (control) groups. No significant differences in protein expression were observed between the treated and control groups in all subsets (Figure 6).

## 4. Discussion

The present study investigated the effect of monocyte differentiation and Mφ polarization on the expression levels of RPS3, RPL13A, RPS15A, and RPL17 in vitro, using the THP-1 monocyte cell line. THP-1 is a human leukemia-derived monocytic cell line model that, after differentiation, displays morphology, functionality, and differentiation markers comparable to primary Mφs [30,31]. This model has been widely employed to investigate the mechanisms, regulatory roles, nutrient transport, and signaling pathways of monocytes and Mφs [32]. THP-1 cells can undergo transfection, express C3 complement and Fc receptors, perform phagocytosis, and differentiate into Mφs when exposed to phorbol esters, including TPA or PMA [33]. Many studies have demonstrated that the THP-1 cell line is a validated model for studying Mφ behavior. These cells exhibit characteristics similar to primary human Mφs, including the expression of key surface markers and the capacity to polarize into M1 and M2 phenotypes [30]. The model is also widely used in atherosclerosis research, as THP-1-derived Mφs display significant oxidative stress and inflammatory responses when exposed to oxidized LDL and have been shown to effectively model foam cell formation, which is critical to atherogenesis [31].

Monocyte-derived Mφs are key players in inflammation, playing an essential role in initiating and advancing atherosclerosis [34]. Mφs are involved in every pathological stage of atherosclerosis, driving disease progression from the initial formation of foam cells in early phases to the advanced stages, where they significantly contribute to the rupture of atherosclerotic plaques [35]. Mφs possess a unique characteristic: plasticity, which enables them to respond precisely to local microenvironmental stimuli. In the context of inflammation, Mφs can either promote the inflammatory response or aid in its resolution during wound and tissue repair. Unpolarized Mφs are referred to as M0-Mφs, whereas the two most extensively studied Mφ phenotypes are the classically activated, pro-inflammatory M1-Mφs and the alternatively activated, anti-inflammatory M2-Mφs [36,37]. In atherosclerosis, M1-like Mφs are primarily located in the shoulder regions of plaques, which are susceptible to rupture, whereas M2-like Mφs are predominantly found in the plaque adventitia [38].

Once monocytes differentiate into Mφs, they express scavenger receptors on their surface, including CD36, SR-AI/II, SR-BI, and LOX-1, enabling recognition of oxidized LDL particles [10]. By engulfing modified LDL particles, these cells are converted into so-called “foam cells,” which are cholesterol ester-laden Mφs and the hallmark of atherosclerotic lesions [39]. Recent studies indicate that PMA concentrations used for differentiating THP-1 monocytes range from 5 ng/mL to 200 ng/mL, with induction periods of either 24 or 48 h [40]. In our study, monocytes were treated with 100 nM PMA for 48 h, and multiple parameters in the differentiated M0-Mφs were assessed to validate our model, consistent with our previous findings [14,29], where similar changes in M0-Mφs compared to monocytes were reported. Following differentiation, M0-Mφs were polarized to the M1 phenotype by treatment with 20 ng/mL IFN-γ and 100 ng/mL LPS. In parallel, M2 polarization was induced by treating M0-Mφs with 20 ng/mL IL-13 and 20 ng/mL recombinant human IL-4, both for 24 h [14,29].

Ribosomal proteins are abundant RNA-binding proteins that serve as key components of the ribosome. They stabilize the rRNA structure within the ribosome, ensuring efficient protein synthesis [41]. Several studies demonstrate that various ribosomal proteins perform extra-ribosomal functions beyond protein synthesis, including crucial roles in gene expression, DNA repair, cell cycle regulation, nucleic acid replication, development, disease, and aging [42]. Thus, while ribosomal proteins are traditionally associated with protein synthesis, they also influence many additional processes linked to the regulation of inflammation. Several ribosomal proteins have been associated with the onset of cardiovascular diseases [43]. In our study, we selected four ribosomal proteins—RPS3, RPL13A, RPS15A, and RPL17—to investigate their potential involvement in Mφ pathobiology using the THP-1 cell model. Our selection was based on previous studies showing that these proteins may be associated with Mφ pathophysiology in various ways [43].

Our results showed no significant change in RPS3 expression in M0- or M2-Mφs compared with monocytes; however, a non-significant trend toward reduced RPS3 expression was observed in M1-Mφs (*p* = 0.059; 0.195-fold decrease). Additionally, RPS15A expression was significantly reduced in M0-, M1-, and M2-Mφs compared with monocytes, corresponding to reductions of 0.384-fold, 0.289-fold, and 0.189-fold, respectively. Recent studies indicate that RPS3 downregulation reduces neurological damage after ischemic stroke; RPS3 knockdown causes microglial polarization from M1 to M2 by suppressing the NLRP3 inflammasome via SIRT1 in vitro [23]. RPS3 expression is also downregulated in peripheral immune cells in response to TLR7 agonists, supporting a role for RPS3 in immune regulation [44]. Similarly, RPS15A has been implicated in venous thromboembolism (VTE) [26], where Mφ polarization and the NLRP3/IL-1/NF-κB pathway are essential in disease development. VTE rats exhibit higher TNF-α and IL-1 levels, increased NLRP3, NF-κB P65, Caspase-1, and IL-1β expression, and a higher proportion of pro-inflammatory M1-Mφs than M2-Mφs [45].

Although our findings do not fully validate an extra-ribosomal role for RPS3 and RPS15A, they link changes in their expression patterns to monocyte differentiation and Mφ polarization, suggesting a potential association with inflammatory processes relevant to cardiovascular disease. Further studies are needed to clarify the pathways through which RPS3 or RPS15A may influence Mφ functions, potentially affecting signaling pathways involved in Mφ polarization such as NF-κB or STAT, which are central to inflammatory responses. Meanwhile, the significant reduction in RPS15A expression was based on three independent biological experiments; therefore, the limited sample size should be considered when interpreting this finding, as it reduces statistical power and increases uncertainty regarding the estimated effect.

RPL13A knockout in Mφs has been shown to induce more severe atherosclerosis in high-fat diet-fed apolipoprotein E–deficient mice, revealing the importance of RPL13A-dependent translational control in protecting against atherosclerosis [24]. In our study, RPL13A expression was unaffected by monocyte differentiation and Mφ polarization, consistent with previous findings identifying RPL13A as a stable housekeeping gene in J774A1 murine Mφs, even under extracellular matrix-stimulating conditions [46]. Similarly, differentiation and polarization did not affect RPL17 expression. Although Smolock et al. (2012) linked RPL17 to reduced vascular smooth muscle growth and carotid intima formation, implying a potential atheroprotective role, our study did not detect a role in THP-1 Mφ pathobiology. This discrepancy may be due to differences in cell models and experimental conditions, as Smolock et al. studied RPL17 in the context of disturbed flow and oxidative stress, whereas our study focused on THP-1 differentiation and polarization [27].

Finally, we assessed the effect of physiological concentrations of Mox-LDL (50 μg/mL) on the expression of RPS3, RPL13A, RPS15A, and RPL17 in THP-1 monocytes and M0, M1, and M2-Mφs to examine the impact of modified LDL on these ribosomal proteins. LDL was oxidized by MPO, which is considered one of the most physiologically relevant oxidation methods compared to alternatives used in previous studies [47,48,49,50]. Mox-LDL has been shown to promote atherogenesis by activating endothelial cells and Mφs to produce pro-inflammatory cytokines and anti-fibrinolytic factors (IL-8, neuroserpin, TNF-α), while reducing IL-10 release and enhancing lipid accumulation in Mφs, thus promoting a pro-inflammatory state, foam cell formation, and plaque progression [13,14,51]. In our study, Mox-LDL did not significantly alter the expression of RPS3, RPL13A, RPS15A, or RPL17 under the tested conditions. Previous studies indicate that RPS3 interacts with p53 under oxidative stress, protecting it from MDM2-mediated degradation and promoting apoptosis [52], suggesting a potential pro-atherogenic role. However, no association between Mox-LDL and RPS3 expression was observed in our model. Our RPL13A expression results are consistent with those of Jia et al. (2012), who reported that 50 µg/mL MPO-oxidized LDL did not significantly affect RPL13A in human monocytes; however, co-treatment with 500 units/mL IFN-γ and 50 µg/mL oxidized LDL completely suppressed RPL13A after 24 h. This may involve S-nitrosylation of GAPDH, which normally interacts with RPL13A to prevent proteasomal degradation, leading to polyubiquitination and degradation of RPL13A [53]. Thus, RPL13A degradation in response to modified LDL may require additional pro-inflammatory stimuli such as IFN-γ. Similarly, Mox-LDL did not affect RPS15A or RPL17 expression in THP-1 monocytes or Mφs under our conditions. Different concentrations, treatment durations, or LDL oxidation methods could potentially alter these results [54] and, thus, should be addressed in future relevant studies. Meanwhile, it should be acknowledged that PMA-induced differentiation is known to trigger broad transcriptional and translational reprogramming, and therefore, global changes in the translation machinery cannot be fully excluded as contributors to the observed alterations in ribosomal protein expression. While our analysis focused on selected ribosomal proteins of interest, we cannot rule out the possibility that PMA treatment may affect ribosomal protein expression more broadly or influence overall translational activity. At present, no comprehensive assessment of global translation or an expanded ribosomal protein panel was performed; however, such approaches, including broader ribosomal protein analyses, would be valuable in future studies to further delineate specific regulatory effects from generalized changes in translational capacity. Another limitation of the present study is the absence of parallel RNA expression analyses to distinguish between transcriptional and post-transcriptional regulation of the ribosomal proteins investigated. Given the well-documented extraribosomal functions of several ribosomal proteins, changes observed at the protein level may reflect regulation occurring at multiple levels. Although complementary mRNA analyses, such as quantitative real-time PCR, were not performed in this study, incorporating transcript-level assessments in future work would provide additional insight into the mechanisms underlying the observed expression changes.

## 5. Conclusions

In conclusion, this study investigated the influence of monocyte differentiation, macrophage polarization, and Mox-LDL treatment on the expression of RPS3, RPL13A, RPS15A, and RPL17 in THP-1 cells. Differentiation and polarization were associated with significant downregulation of RPS15A, whereas RPS3 showed a non-significant trend toward reduced expression in M1 macrophages, and no significant changes were observed for RPL13A or RPL17. Under the experimental conditions examined, Mox-LDL treatment was not associated with significant changes in the expression of the investigated proteins. While preliminary and limited to a THP-1 cell-line model, these findings identify selective alterations in ribosomal protein expression during monocyte-to-macrophage differentiation and macrophage polarization. Further studies, particularly in primary human monocytes/macrophages and using functional approaches, are warranted to validate these observations, determine their biological significance, and assess their potential relevance to atherosclerosis.

## Data Availability

All data generated or analyzed during this study are included in this article and its Supplementary Materials.

## Supplementary Materials

The following supporting information can be downloaded at: https://www.mdpi.com/article/10.3390/jcdd13090450/s1, Supplementary Figure S1: CD11b Surface Expression on THP-1 Monocytes and M0-Mφs. (a) Representative histogram plots and (b) bar graphs demonstrating the surface expression of CD11b on THP-1 monocytes and M0-Mφs. Column bars represent mean values of the percentage (%) or geometric fluorescent intensity (MFI) of CD11b positive (+ve) cells from 3 independent experiments. Error bars represent SEM. Unpaired *t*-test was used to calculate statistical significance. ** *p* < 0.01 and **** *p* < 0.0001 versus THP-1 monocytes. Supplementary Figure S2: CD11c Surface Expression on THP-1 Monocytes and M0-Mφs. (a) Representative histogram plots and (b) bar graphs demonstrating the surface expression of CD11c on THP-1 monocytes and M0-Mφs. Column bars represent mean values of the percentage (%) or geometric mean fluorescent intensity (MFI) of CD11c positive (+ve) cells from 3 independent experiments. Error bars represent SEM. Unpaired *t*-test was used to calculate statistical significance. ** *p* < 0.01 and *** *p* < 0.001 versus THP-1 monocytes. Supplementary Figure S3: Cell Surface Marker Expression of CD80 and CD209 on the Different THP-1 Derived Mφ Types. (A) Column bars represent mean values of the percentage (%) and geometric mean fluorescence intensity (MFI) of CD80^+^ THP-1 derived Mφs from 3 independent experiments. (B) Column bars represent mean values of the percentage (%) and geometric mean fluorescence intensity (MFI) of CD209^+^ THP-1 derived Mφs from 3 independent experiments. Error bars represent SEM. Statistically significant differences were determined by one-way ANOVA followed by Tukey’s multiple comparison post hoc test (* *p* < 0.05; ** *p* < 0.01).
